# Cemented versus uncemented hemiarthroplasty for femoral neck fractures in patients with neuromuscular diseases: a minimum of 2 years’ follow-up study

**DOI:** 10.1186/s13018-021-02572-6

**Published:** 2021-07-01

**Authors:** Yuchuan Wang, Zhongzheng Wang, Siyu Tian, Zhanchao Tan, Yanbin Zhu, Wei Chen, Yingze Zhang

**Affiliations:** 1 Department of Orthopaedic Surgery, The 3rd Hospital of Hebei Medical University, Shijiazhuang, 050051 Hebei People’s Republic of China; 2 Orthopaedic Institution of Hebei Province, Shijiazhuang, 050051 Hebei People’s Republic of China; 3 Key Laboratory of Biomechanics of Hebei Province, Shijiazhuang, 050051 Hebei People’s Republic of China; 4 NHC Key Laboratory of Intelligent Orthopeadic Equipment, Shijiazhuang, 050051 Hebei People’s Republic of China; 5 Chinese Academy of Engineering, Beijing, 100088 People’s Republic of China

**Keywords:** Femoral neck fracture, Neuromuscular disease, Cemented hemiarthroplasty, Uncemented hemiarthroplasty

## Abstract

**Background:**

The aim of this study was to compare the outcomes of cemented and uncemented hemiarthroplasty for femoral neck fractures in patients with neuromuscular disease.

**Methods:**

We reviewed 156 patients with neuromuscular disease who underwent hemiarthroplasty between June 2015 and December 2019. Patients were divided into cemented group (*n* = 105) and uncemented group (*n* = 51), with a minimum follow-up of 2 years. Factors including preoperative features, duration of surgery, intraoperative blood loss, complications, pain, Harris hip scores (HHS), and quality of life were compared across groups, and Kaplan–Meier curves were used to estimate survival.

**Results:**

In the uncemented group, the mean duration of surgery was 16.0 min. shorter (*p* = 0.001) and the mean intraoperative blood loss was 71.1 mL less (*p* = 0.01). Visual analog scales (VAS), HHS, and European Quality of Life-5 Dimensions (EQ-5D) scores were not different between the groups. Despite a few potential trends, we did not observe a difference in complications such as periprosthetic fractures and dislocations. The rates of mortality were similar between groups (*p*=0.821).

**Conclusions:**

Both arthroplasties may be used with good medium-term results in the treatment of femoral neck fractures in patients with neuromuscular diseases.

## Introduction

Evidences suggested patients with neuromuscular disease have an increased risk of falls and osteoporosis, which increases the risk of femoral neck fractures in these patients [1, 2]. The hemiarthroplasty has been considered to be the most cost-effective treatment option for displaced femoral neck fractures in the elderly and commonly used as a preventive measure for dislocation [3]. This is especially the case in patients with neuromuscular defects secondary to dementia, cerebrovascular accident, poliomyelitis, or Parkinson’s disease [4]. Many patients with neuromuscular disease eventually require hemiarthroplasty because of femoral neck fractures, but no definite conclusions have been made regarding which type of hemiarthroplasty is preferred.

For the treatment of femoral neck fractures in patients without neuromuscular disease, some studies showed that cemented hemiarthroplasty was associated with lower risk of periprosthetic fracture and revision surgery, as well as higher levels of function and patient satisfaction [5–7]. However, some surgeons prefer to apply the uncemented hemiarthroplasty technique, because they believe it can reduce duration of surgery, intraoperative blood loss, perioperative mortality, and risk of bone cement implantation syndrome (BCIS) [8–10]. Previous studies usually regarded preoperative neuromuscular conditions as a variable affecting dislocation, reoperation, and other complications after hemiarthroplasty, and focused on the relationship between them [11, 12], we are unaware of any randomized trials comparing hemiarthroplasties using cemented implants with uncemented implants for treatment of femoral neck fractures in patients with neuromuscular disease.

It is unclear whether cemented hemiarthroplasty could yield the same clinical results as uncemented hemiarthroplasty in the treatment of displaced femoral neck fractures in patients with neuromuscular diseases. The purpose of this study therefore was to compare the clinical outcomes associated with cemented versus uncemented hemiarthroplasty for femoral neck fractures in patients with neuromuscular diseases.

## Methods

**Fig. 1:**
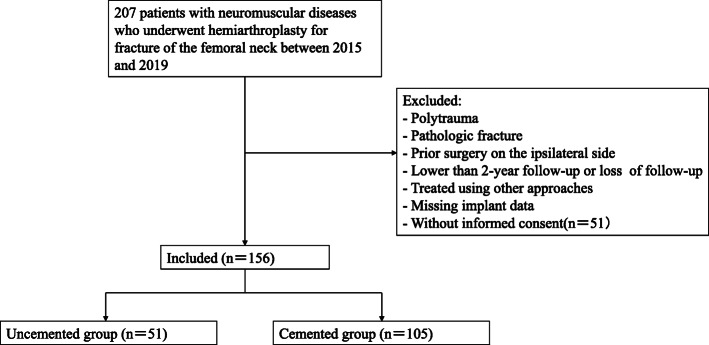
Flowchart showing the data collection methodology and excluded cases

Between June 2015 and December 2019, 207 patients with neuromuscular disease were surgically treated using a primary hemiarthroplasty. Patients with unilateral displaced femoral neck fracture, posterolateral approach, and follow-up for at least 2 years were included in the study. Cases were excluded if they involved polytrauma, pathologic fracture, prior surgery on the ipsilateral side, less than 2-year follow-up, or loss of follow-up, or were treated using other approaches or lack of implant data (Fig. 1). A total of 156 consecutive patients were finally enrolled. All patients who can provide informed consent did so. Patients who were unable to give their informed consent due to cognitive impairment were included if it was considered to be in their best interest after consultation with their family members. Also excluded were patients with consciousness disorders from whom the consent of the next of kin could not be obtained. The study was approved by the Ethics Committee of the Third Hospital of Hebei Medical University, according to the Helsinki Declaration.

Patients with an uncemented implant for hemiarthroplasty were assigned to uncemented group (*n*=51) and those with a cemented implant were assigned to cemented group (*n*=105). All operations were carried out using a posterolateral approach to the hip in the lateral decubitus position. After surgery, all patients were advised to avoid excessive flexion, internal rotation, and adduction in hip movements. In cases where general conditions allowed it, early mobilization was encouraged in all patients with weight-bearing as tolerated in both groups.

Data on patient demographics, medical comorbidities, procedure details, and perioperative complications were collected from the electronic medical record by a trained research associate who was blinded to the treatment allocation. All patients were invited to the outpatient clinic for follow-up. Those who were unwilling or unable to attend follow-up visits were interviewed by telephone. The factors assessed were duration of surgery, intraoperative blood loss, perioperative and postoperative complications, pain, functional outcomes, quality of life at last follow-up, and survivorship. The VAS scale was used to assess postoperative pain, with responses ranging from 0 to 10. Hip function was rated using the HHS [13], ranging from 0 to 100 points and covering a maximum of 44 points for painless, 47 points for function, and 9 points for range of motion and absence of deformity. Health-related quality of life (HRQoL) was rated by EQ-5D score. We used the EQ-5D index score, which ranged from 0 (worst health) to 1 (perfect health), as well as the EQ-5D visual analog scale (EQ-VAS) ranging from 0 (worst possible health) to 100 (best possible health).

Mean, standard deviation, lowest and highest values, and frequency ratio were used in the descriptive statistics of data. Categorical variables were compared using chi-squared test and continuous variables were compared using independent-samples *t*-test. Patient survival was determined by Kaplan–Meier curves. The log-rank test was used to determine whether there was a difference in survival curves. All statistical analyses were recorded and analyzed using SPSS, version 22.0, for Windows (SPSS Inc., Chicago). Statistical significance was set at a *p*-value < 0.05.

## Results

The mean follow-up time was 40 months (range, 24–74 months). No difference was detected in terms of gender, age, side of fracture, American Society of Anesthesiologists scores (ASA), or underlying diseases between the two treatment groups (Table 1). However, the uncemented group had significantly shorter operation time and less intraoperative blood loss. The mean operation time in the uncemented group was 102.3 ± 28.3 min compared with 118.3 ± 28.7 min in the cemented group (*p*=0.001), and the average intraoperative blood loss was 299.0 ± 133.2 mL in the uncemented group compared with 370.1 ± 171.2 mL in the cemented group (*p* = 0.01).

**Table 1:** Baseline characteristics and postoperative complications

Variable	Uncemented (*n*=51)	Cemented (*n*=105)	*p* value
Sex			0.138
Female	24	66	
Male	27	39	
Average age at surgery (Range; SD)	72.6 (53–87; ± 8.7)	75.1 (51–93; ± 8.4)	0.078
Side			0.279
Left	22	55	
Right	29	50	
ASA preoperative risk score			0.524
1	11	16	
2	18	38	
3	16	30	
4	6	21	
Underlying disease			0.865
Stroke	30	67	
Parkinsonism	8	18	
Dementia	7	11	
Epilepsy	3	5	
Poliomyelitis	3	3	
Myasthenia gravis	0	1	
Postoperative complications			
Pneumonia	1	7	0.211
Congestive cardiac failure	1	1	0.599
Cardiac arrhythmia	1	0	0.150
Deep vein thrombosis	3	7	0.851
Dislocation	4	3	0.158
Periprosthetic fracture	3	1	0.068

*n*, sample number; *SD*, standard deviation; *ASA*, American Society of Anesthesiologists scores

The number of postoperative periprosthetic fractures was three (5.9%) in the uncemented group and one (1.0%) in the cemented group (*p*=0.068). We found a total incidence of dislocation of 4.5% (seven of 156). Among these 156 patients, the incidence of dislocation was 7.8% (four of 51) in the uncemented group and 2.9% (three of 105) in the cemented group (*p*=0.158). In addition, 21 patients (13.5%) developed perioperative medical complications, 15 in the cemented group and 6 in the uncemented group (Table 1). No patient required further surgery due to infection. There was no difference in general or local complications between the two groups.

None of the three pain and functional outcome scales, VAS, HHS, and EQ-5D, showed any differences between groups (Table 2).

**Table 2:** Pain and functional outcomes

Variable	Uncemented (*n*=51)	Cemented (*n*=105)	*p* value
Harris hip score*	76.4 ± 15.9	71.8 ±14.1	0.105
EQ-5D index score*	0.6 ± 0.3	0.6 ± 0.2	0.719
EQ-5D visual analog scale*	75.6 ± 15.8	69.2 ± 14.4	0.126
VAS pains scale*	0.6 ± 1.1	0.7 ± 1.2	0.668

*n*, sample number; *VAS*, visual analog scale; *EQ-5D*, European Quality of Life-5 Dimensions

*Values are given as means ± standard deviation

**Fig. 2:**
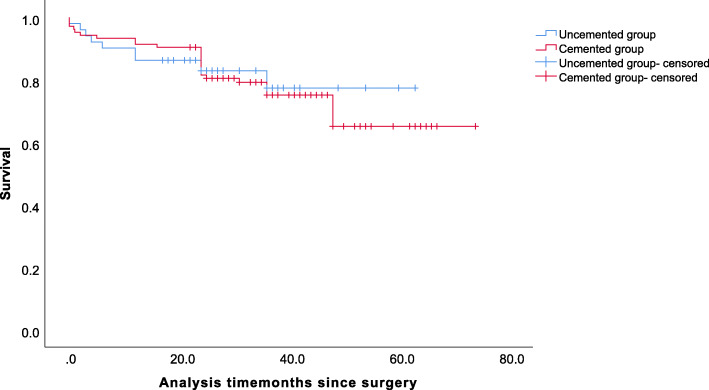
Kaplan–Meier curve with mortality as the endpoint for cumulative survival according to group

Finally, there was no intraoperative death. Kaplan–Meier analysis revealed a mean survival of 52.3 months (95% CI 46.0 to 58.7) for the uncemented group and 57.7 months (95% CI 52.6 to 62.9) for the cemented group (Fig. 2), but the difference was not statistically significant (*p* = 0.821). There was no difference in mortality between the two groups (*p* = 0.821), with a 4-year survival rate of 65.1% (95% CI 53 to 77) in the cemented group and 77.4% (95% CI 63 to 92) in the uncemented group (Table 3).

**Table 3:** Kaplan–Meier survival with 95% CI (mortality as endpoint)

	Survival (%)				
	3 months	6 months	12 months	24 months	48 months
Uncemented (95% CI)	94.1 (88–99%)	90.2 (82–98%)	86.3 (77–96%)	83.0 (72–94%)	77.4 (63–92%)
Cemented (95% CI)	94.3 (90–99%)	93.3 (89–98%)	91.4 (86–97%)	81.5 (74–89%)	65.1 (53–77%)

*CI*, confidence interval

## Discussion

To our knowledge, this is the first consecutive cohort study to compare the relative clinical outcomes of cemented and uncemented hemiarthroplasty in the treatment of femoral neck fractures in patients with neuromuscular disease. This retrospective case-control study demonstrated that the uncemented hemiarthroplasty had shorter duration of surgery and less intraoperative blood loss, but the two procedures for patients with neuromuscular diseases were equally good regarding functional outcomes, health-related quality of life, mortality, or general and local complications.

There were several advantages in the use of uncemented implants, which were used in our study. The amount of duration of surgery and intraoperative blood loss can be reduced in uncemented implants compared with cemented implants, which will be partly due to the time for the polymerization of the cement. Ng and Krishna recommended that uncemented hemiarthroplasty was preferred over cemented hemiarthroplasty because of reduced duration of surgery and intraoperative blood loss [14]. Figved and colleagues conducted a two-center randomized equivalence trial involving 230 patients in New Zealand and found the duration of surgery and intraoperative blood loss were less in the uncemented group [15]. Many of patients with neuromuscular diseases have complex deformities and extensive contractures, which makes them more challenging surgery [16], and as a result, the duration of surgery of these patients may be longer than that of patients without neuromuscular disease, and the intraoperative blood loss may be more. In addition, in our study, part of the reason for the increase of uncemented hemiarthroplasty in recent years may be that cemented fixation takes more time, and cement removal can be difficult if a revision surgery is required in the future. The shorter operative times and less intraoperative blood loss may have some organizational and economic benefits, but should not be overestimated.

Notably, none of the three pain and functional outcome scales, VAS, HHS, and EQ-5D, showed any differences between the groups in our study. Similar studies have also shown that in patients without neuromuscular disease, there were no differences in pain or patient-reported outcome measures at 1 year [17, 18]. Parker and colleagues found cement to be associated with less pain and better mobility and Inngul et al. found better outcomes as measured by the Short Musculoskeletal Functional Assessment, HHS, and EQ-5D scores for the cemented implant [5, 19]. However, our results suggested that cemented hemiarthroplasty cannot reduce the risk of pain and improve functional outcomes in patients with neuromuscular disease. As life expectancy increases for patients with neuromuscular disease, these patients will have higher expectations for mobility and quality of life. We found the cemented and uncemented implants used in our study were equally good in terms of the functional outcome and health-related quality of life.

Although the between-group difference was not statistically significant, we found that the uncemented group was associated with higher risk of postoperative periprosthetic fracture (5.9% in the cemented group vs. 1.0% in the uncemented group), which is supported by previous studies. Barenius and colleagues conducted a randomized trial comparing 67 cemented prostheses with 74 uncemented prostheses and found 6.8% periprosthetic fractures in the uncemented group, compared with 3% in the cemented group [20]. Similarly, Morris and colleagues found 5 periprosthetic fractures in the uncemented group (10.7 %) as compared to none in the cemented group [21]. The specific reason for the improved outcome observed in patients with cemented hemiarthroplasty has not been definitively clarified. Patients with neuromuscular diseases are well known to be at significant risk of osteoporosis [22], and imbalances in muscle strength may lead to an increased risk of falls, all of which may cause an increased incidence of periprosthetic fractures in such patients. One theory is that cemented fixation may be better resistant to periprosthetic fracture in patients with risk factors such as neuromuscular diseases, a history of falls, and osteoporosis [23]. Although our results showed no significant difference in periprosthetic fractures between groups, we should be cautious about this complication. Large sample studies may be needed to assess whether the implants used in our study have a different risk of periprosthetic fracture.

Dislocation of a hemiarthroplasty is uncommon, with an incidence of 1.5 to 2.0% [24–26]. Evidences also suggested patients with neuromuscular diseases had a higher incidence of dislocation after hemiarthroplasty than those without, ranging from 4.8 to 45% [26–28]. The total incidence of dislocation in our study was 4.5%, slightly lower than in previous studies, with a dislocation rate of 7.8% in the uncemented group and 2.9% in the cemented group, with no significant difference between the two groups. Cognitive dysfunction from dementia, psychosis, or confusion is a reported risk factor for hip instability and neuromuscular dysfunction positively correlates with dislocation [29, 30]. However, Suh et al. found no difference in the incidence of dislocation between patients with or without neuromuscular disease by using a posterior soft tissue repair technique to maintain adequate soft tissue tension [28]. Some have suggested that the posterior approach is a risk factor for dislocation after hemiarthroplasty [31–33], but others have shown that surgical approach and dislocation do not correlate after hemiarthroplasty [26, 28, 34]. Because all of patients in our study were operated with posterolateral approach, comparison with another approach is needed. Based on our results and previous studies, patients with neuromuscular disease have a higher risk of dislocation than the general population, and both arthroplasties were appropriate.

In addition to periprosthetic fracture and dislocation, there are other complications such as pneumonia and deep vein thrombosis, which may be related to the fact that patients with neuromuscular diseases are less likely to follow the postoperative procedures and guidelines to facilitate rapid recovery without complications [35]. But there were no significant differences in these complications. Interestingly, the number of cardiovascular complications did not differ between the groups, contrary to the widely held view that there is an association between cement and cardiovascular complications [36].

Intraoperative mortality is the most worrying complication for patient, their family, and surgeon. Intraoperative death almost exclusively occurred during cemented procedures, which may be caused by the BCIS [37], but no intraoperative death was found in our study. The Kaplan–Meier curve was performed to analyze patients’ survivorship and the result showed distinct similarity among the two groups, which was comparable with previous studies. Fenelon et al. found no difference in mortality between cemented and uncemented hemiarthroplasty at 7 days, 30 days, and 1 year [38]. Similarly, we believe that the use of cement has no detrimental effect on the short- and mid-term mortality in patients with neuromuscular diseases.

Several limitations must be considered when interpreting the presented data. First, this was a retrospective study with limited patient numbers. Future high-quality studies with larger sample size and longer follow-up are warranted to confirm the results of our study. Second, patients did not undergo conventional dual-energy x-ray absorptiometry testing, so it is impossible to exclude the difference of bone mineral density, which could affect the risk of periprosthetic fracture. However, in our study, all patients developed a low-energy hip fracture, which met the clinical criteria for osteoporosis by definition. Third, some patients have dementia and cannot participate in the extensive follow-up, so we can only obtain the required follow-up score of such patients through their family members, which may have added a risk of bias. Since the scores were obtained after communicating with family members as fully as possible, the risk of bias is assumed to be limited.

## Conclusion

In summary, our results reflect that an uncemented implant offers functional outcomes, health-related quality of life, and mortality to equal those of a cemented implant. Despite a few potential trends, we did not observe a difference in complications such as periprosthetic fractures and dislocations. The seeming advantages of less intraoperative blood loss and shorter duration of surgery of uncemented hemiarthroplasty are of little importance compared with the important findings of equivalent functional results. Both arthroplasties may be used with good medium-term results in the treatment of femoral neck fractures in patients with neuromuscular diseases.

## Data Availability

The data and materials contributing to this article may be made available upon request by sending an e-mail to the corresponding author.

## Abbreviations

HHS: Harris hip scores
VAS: Visual analog scales
EQ-5D: European Quality of Life-5 Dimensions
BCIS: bone cement implantation syndrome
HRQoL: Health-related quality of life
ASA: American Society of Anesthesiologists scores
